# Posterior circulation cerebral infarction: A review of clinical, imaging features, management, and outcomes

**DOI:** 10.1016/j.ejro.2023.100523

**Published:** 2023-09-14

**Authors:** Rashid A. Ahmed, Adam A. Dmytriw, Robert W. Regenhardt, Thabele M. Leslie-Mazwi, Joshua A. Hirsch

**Affiliations:** a Department of Neurology, Massachusetts General Hospital, Harvard Medical School, USA; b Department of Neurosurgery, Massachusetts General Hospital, Harvard Medical School, USA; c Department of Radiology, Massachusetts General Hospital, Harvard Medical School, USA; d Department of Neurology, Neurosciences Institute, University of Washington, Seattle, WA, USA

**Keywords:** Posterior circulation stroke, Endovascular therapy, Basilar artery occlusion, Neuroimaging

## Abstract

**Objective:**

This narrative review discusses posterior circulation cerebral infarcts (PCCI) and provides an update given recent randomized trials in the management of basilar artery occlusion (BAO). We examine clinical characteristics, imaging protocols, management updates, and outcomes of PCCI.

**Methods:**

The following databases were searched: MEDLINE, Scopus, Google Scholar, and Web of Science for articles on PCCI. We included randomized trials and observational studies in humans. We also reviewed relevant references from the literature identified.

**Results:**

PCCI and BAO is associated with high morbidity and mortality. Early assessment and accurate diagnosis of PCCI remains a clinical challenge. Neuroimaging advances have improved early detection, but barriers remain due to costs and availability. Recent randomized trials provide new insights for BAO patients and support the efficacy of endovascular thrombectomy.

**Discussion:**

PCCI requires specific diagnostic and management that is distinct from anterior circulation stroke. While further studies are needed in varied populations and in the subset of BAO patients presenting with milder deficits, growing randomized data support the treatment of BAO patients with endovascular thrombectomy.

## 1 Introduction

Posterior circulation cerebral infarction (PCCI) includes ischemia to brain territories supplied by the posterior cerebral arteries, basilar artery, intracranial, and extracranial vertebral arteries. PCCI accounts for approximately 26 % of all ischemic strokes. The commonest regions involved include the brainstem, cerebellum, occipital lobe, and medial temporal lobe [1]. Common risk factors include hypertension, smoking, alcohol consumption, diabetes mellitus, hyperlipemia, coronary artery disease, and atrial fibrillation. The commonest etiology reported for PCCI varies by study and geographical region. In an observational study performed in China, PCCI was primarily noted to be from small vessel disease (37.6 %), large artery atherosclerosis (29 %), or cardioembolism (5.4 %) [2]. In the New England Posterior Circulation Registry, which included 407 patients, the commonest stroke etiology was embolism (with primary origins 24 % cardiac and 14 % proximal vessel disease), followed by large artery occlusive lesions (32 %) and branch artery occlusion (14 %) [3]. An important etiology in younger patients is vertebral artery dissection which accounts for up to 25 % of PCCI in this population [4].

With advances in imaging and treatment, the overall mortality and long-term disability associated with PCCI has improved. In this review, we summarize the clinical presentation, imaging, management, and outcomes of PCCI.

## 2 Methods

The following databases were searched: MEDLINE, Scopus, Google Scholar, and Web of Science. The following search terms were used: “posterior circulation stroke,” “posterior circulation stroke and clinical presentation,” “posterior circulation stroke and imaging,” “posterior circulation stroke and medical therapy,” “posterior circulation stroke and mechanical thrombectomy,” “posterior circulation stroke and endovascular thrombectomy,” and “posterior circulation stroke and outcomes.” We included randomized trials and observational studies in humans. We also reviewed relevant references from the literature identified. Studies were excluded if they met predetermined exclusion criteria, including preclinical studies or those involving animal models of stroke.

## 3 Clinical presentation

The classic clinical presentations for PCCI are the “5 Ds”; dysphagia, diplopia, dysarthria, dizziness, and dystaxia. The cerebrovascular anatomy of the posterior circulation can lead to highly specific signs in PCCI. Signs such as internuclear ophthalmoplegia, vertical nystagmus, conjugate paresis to one side, unilateral palsy of eye movements, and skew deviation are pathognomonic for brain stem PCCI [5]. Nausea, vertigo, conjugate nystagmus in any direction, and ataxia are characteristic of cerebellar and brainstem PCCI. The bilateral blood supply of the basilar artery to the posterior fossa and brain stem fiber tract crossing leads to highly specific crossed syndromes and bilateral long tract signs. Upper brainstem and cerebellar lesions lead to gait ataxia and unsteadiness, while lower brainstem lesions tend to cause true vertigo and conjugate horizontal/rotatory nystagmus. A decreased level of consciousness and amnestic syndromes can also be indicative of PCCI. Involvement of the thalamus can lead to aphasia, and cerebellar strokes can cause cognitive deficits due to interactions of cerebrocerebellar circuitry [6], [7], [8], [9].

Unilateral limb weakness (38 %) and gait ataxia (31 %) were the commonest signs [7]. In the Chengdu Stroke Registry, Horner’s syndrome, quadrantanopia, oculomotor nerve palsy, crossed sensory deficits, and crossed motor deficits had the highest predictive values favoring a diagnosis of PCCI. It is important to note that the sensitivity of these signs was very low ranging from 1.3 % to 4.0 % [8]. Highly specific signs are not always present, however. Dizziness (47 %), dysarthria (31 %), unilateral limb weakness (41 %), nausea or vomiting (27 %), and headache (28 %) were the most frequent symptoms of PCCI noted in the New England Posterior Circulation Registry [7].

Recognizing PCCI can be challenging given this variable presentation. Furthermore, PCCI can present with variable temporal course, with either abrupt or stuttering symptoms. Some patients have prodromal symptoms for up to 2 weeks prior to a posterior circulation large vessel occlusion. Conditions that can mimic PCCI are variable and broad. These may include intracranial hemorrhage, subarachnoid hemorrhage, basilar migraine, toxic or metabolic disturbances, central pontine myelinolysis, Miller Fisher syndrome, posterior reversible encephalopathy syndrome, neuroinflammatory disorders (Behcet’s disease, Whipple’s disease, sarcoidosis), and rhombencephalitis [10]. For these reasons, there are often delays in PCCI diagnosis, and it is essential to maintain a high index of suspicion.

Numerous prehospital acronyms have been developed for pre-hospital stroke assessment, the commonest being The Face Arm Speech Test (FAST). This mnemonic, adopted by the American Heart Association, is commonly used in public education campaigns, as well as emergency medical services in some regions. The FAST test has been shown to miss 40 % of PCCI as compared to only 10 % in the anterior circulation stroke (ACS) [11]. To improve PCCI identification, some educational programs have added a “B” for balance and an “E” for eyes to the FAST mnemonic. In a retrospective study that included 736 patients, the proportion of acute strokes missed using FAST alone was reduced to 4.4 % from 14 % with the addition of gait and visual symptoms (BE-FAST). The proportion of PCCI missed was reduced to 43 % from 71 % with BE-FAST on review of magnetic resonance imaging reports [12]. The majority of the published pre-hospital scales are not effective in the identification of PCCI, and their performance is usually assessed for ACS [13]. In addition, PCCI is three times more likely to be missed in the emergency department as compared to ACS, especially when patients present with vomiting/nausea, dizziness, and a stroke history [14]. Although the National Institutes of Health Stroke Scale (NIHSS) is the commonest scale used to measure the severity of acute ischemic infarcts, it underestimates the severity of deficits in PCCI. This is important in consideration for eligibility for reperfusion therapies. The posterior NIHSS (POST-NIHSS) was developed to improve prognostic accuracy for PCCI in patients presenting with mild, moderate symptoms. Points added to the NIHSS included dysphagia (4 points), abnormal cough (5 points), and gait/truncal ataxia (3 points). The POST-NIHSS was shown to have a higher prognostic accuracy than NIHSS for PCCI but has yet to find widespread utilization [15].

## 4 Imaging

Imaging in PCCI is key given its nonspecific clinical presentation. Non-contrast computed tomography (NCCT) is usually the first-line imaging study obtained in PCCI. The sensitivity of detecting PCCI with NCCT was noted to be 41.8 % by Hwang et al. Beam hardening artifacts and the slow temporal evolution of PCCI are likely responsible for this low sensitivity [16]. Given these limitations, Magnetic Resonance Imaging (MRI) with Diffusion-Weighted Imaging (DWI) is the preferred imaging modality to rule out PCCI. Although DWI is more sensitive in detecting PCCI, cost and availability may limit its use in many real-world settings [17]. Obtaining computed tomographic angiography (CTA) of the head and neck is the typical current standard in ruling out vertebrobasilar occlusion and assessing both intracranial and extracranial vessels. CTA also helps in assessing posterior circulation collaterals and thrombus burden [18], [19]. Although computed tomography perfusion (CTP) has shown promise in predicting outcomes in patients presenting with basilar artery occlusion (BAO), there is a lack of validated thresholds in assessing the ischemic core and penumbra [20], [21], [22].

Several scoring systems have been established to predict outcomes in PCCI. Key factors included in these scoring systems include the location of the clot, collateral status, ischemic changes, and clot burden. Pretreatment Collateral Score (CS), Posterior Circulation CT Angiography (pc-CTA), Basilar Artery on Computed Tomography Angiography (BATMAN), Posterior Circulation Acute Stroke Prognosis Early CT Score (pc-ASPECTS), and Posterior Circulation Collateral Score (PC-CS) are the CTA scoring systems [18], [19], [23], [24], [25], [26]. PC-ASPECTS (which can be used on either modality), Bern DWI, Brainstem DWI, and Renard DWI are the MRI scoring systems [27], [28], [29], [30]. A good 90-day outcome (modified Rankin Scale, mRS ≤3) was noted in 52 % of patients with pc-ASPECTS >8 when compared to 4 % in those with pc-ASPECTS <8 [24]. Poor outcomes among patients treated with endovascular therapy has been reported in patients with BATMAN <7 [18]. Generalization of these scores is challenging given the retrospective nature of the studies, variable outcome definitions, and small sample sizes [31]. In theory, MRI studies could be used to identify the absolute validity of scores before spending time and effort optimizing issues with beam-hardening and other artifacts which limit posterior fossa gray-white resolution on CT.

## 5 Management

### 5.1 Thrombolysis

Comparable safety and efficacy have been demonstrated in observational studies of patients with anterior versus posterior circulation stroke treated with intravenous tissue-type plasminogen activator (IV tPA). A greater proportion of white matter, lower infarct volume, and collaterals might enhance tolerance to ischemic injury, explaining the lower risk of hemorrhagic transformation in PCCI when compared to ACS [32], [33]. PCCI has been underrepresented in the major IV tPA trials. Only 5 % of patients in the National Institute of Neurological Disorders and Stroke (NINDS) study, no patients in the European Cooperative Acute Stroke Study (ECASS) I / II trials, and 8 % of patients in the Third International Stroke Trial [IST-3] suffered from PCCI [34], [35], [36], [37]. Given the nonspecific symptoms and delayed diagnosis, administration of IV tPA is often slower in patients with PCCI when compared to ACS [38].

### 5.2 Endovascular therapy

BAO makes up approximately 1 % of all ischemic strokes and 10 % of large vessel occlusions (LVO) [39]. However, it can be devastating and may lead to poor outcomes in 80 % of patients in the pre-endovascular therapy(EVT) era [40]. For several years, there has been clear evidence supporting the efficacy of EVT over medical treatment in anterior circulation LVOs [41]. This has not been the case with BAO, but there is currently a potential turning point given two recent trials, ATTENTION (Endovascular Treatment For Acute Basilar Artery Occlusion: A Multicenter Randomized Clinical Trial) and BAOCHE (Basilar Artery Occlusion Chinese Endovascular). There were two prior trials, BEST (Basilar Artery Occlusion Endovascular Intervention versus Standard Medical Treatment) and BASICS (Basilar Artery International Cooperation Study), which failed to show a clear benefit of EVT over medical treatment. Despite failing to show a clear efficacy of EVT over medical treatment, these two trials set the stage for establishing power calculations and anticipating adverse events in trials involving BAO patients [42].

BASICS was a multicenter open-label international trial that randomized 300 patients (154 in the EVT arm and 146 in the medical arm) who presented within 6 h from the last known normal. Approximately 80 % of patients in both arms received IV tPA. There was no significant difference in the favorable functional outcome (defined as mRS of 0–3) or symptomatic intracranial hemorrhage (sICH) between the two groups. Selection bias was a concern in this trial as 124/424 patients (29 %) of eligible patients were not enrolled, and 98/124 (79 %) of those not enrolled underwent EVT. Protocol changes during the trial and low patient recruitment were also concerns [43]. Similar results were found in the BEST trial. This was a multicenter, randomized, open-label trial that enrolled 131 patients (66 patients in the EVT arm and 65 patients in the medical arm) within 8 h from the last known normal. There was no difference in the proportion with mRS ≤3 at 90 days comparing the two groups in the intention-to-treat analysis. However, prespecified secondary analysis of the primary outcome showed higher rates of mRS 0–3 at 90 days in the EVT arm when compared to those who received medical therapy. Five patients (8 %) in the EVT arm had sICH compared to none in the medical arm. The trial was terminated early due to poor recruitment and high crossover rates. Selection bias was also a concern, as a third of patients who were eligible for enrollment declined participation [44].

ATTENTION was a multicenter, prospective, randomized trial that enrolled 340 patients (226 in the EVT arm and 114 in the medical arm) within 12 h from symptom onset. Only one-third of patients in both arms received IV- tPA. At 90 days, 46 % of patients in the EVT arm had good functional status (defined as an mRS of 0–3) as compared to 23 % in the medical arm. sICH occurred in 12 patients (5 %) in the EVT arm and none in the medical arm. 90-day mortality was also lower in the EVT arm (37 %) as compared to the medical arm (55 %) [45]. Another trial, BAOCHE, enrolled 217 patients (110 patients in the EVT arm and 107 in the medical arm) between 6 and 24 h after symptom onset. Enrollment was halted after a prespecified interim analysis showed the superiority of EVT. At presentation, 14 % in the EVT arm were treated with IV tPA as compared to 21 % in the medical arm. At 90 days, 46 % of patients in the EVT group had an mRS of 0–3, as compared to 24 % in the medical arm. sICH occurred in 6 patients (6 %) in the EVT arm as compared to 1 patient in the medical arm. The mortality rate at 90 days was 31 % in the EVT arm as compared to 42 % in the medical arm [46]. The crossover rates in both BAOCHE and ATTENTION were limited. Generalizing the results of ATTENTION and BAOCHE is challenging as both trials only enrolled Chinese patients who traditionally have a higher prevalence of intracranial atherosclerosis. Patients with milder deficits were also not well represented in both trials. In ATTENTION, patients with NIHSS <10 were excluded from the study. Although BAOCHE did enroll patients with at least an NIHSS of 6, 92 % of patients enrolled had a baseline NIHSS of at least 10.

It is important to note that the pc-ASPECTS in both BAOCHE (median score of 8 in both groups) and ATTENTION (9 in the EVT arm and 10 in controls) were on the higher side since patients with significant evidence of infarction were excluded. Also, the percentage of patients who got IV tPA in BAOCHE (18 %) and ATTENTION (32 %) is lower when compared to BASICS (79 %). The likely reasons for these differences include the later window criteria for enrollment and the costs that were required from patients in BAOCHE and ATTENTION. All the BAO randomized trials performed in China showed a higher prevalence of intracranial atherosclerosis. This necessitated treating patients in the EVT arm with stenting or angioplasty. In addition, collaterals may have been enhanced with chronic high-grade basilar artery stenosis increasing resistance to ischemia. Lastly, the sICH incidence in the medical arm in BAO trials has been ≤1 % when compared to anterior circulation trials (∼ 4 %). This may support the argument for using intravenous thrombolysis in the extended time window in patients with BAO [42]. This is currently being evaluated in the Extending the Time Window for Tenecteplase by Recanalization of Basilar Artery Occlusion in Posterior Circulation Stroke (POST-ETERNAL). Patients presenting to the emergency department with BAO within 24 h of stroke onset will receive either intravenous tenecteplase (0.25 mg/kg, maximum 25 mg, administered as a bolus over 5–10 seconds) or standard of care (alteplase 0.9 mg/kg or no lysis) + /- EVT.(ClinicalTrials.gov number, NCT05105633) [42], [43], [44], [45], [46]. A meta-analysis that included aggregate data from all 4 BAO randomized trials (BASICS, BEST, BAOCHE, ATTENTION) revealed that EVT correlated with improved outcomes when compared with medical therapy. The pooled RR for 90-day mRS 0–3 was 1.54 (95 % CI 1.16–2.04; heterogeneity I2 = 60 %). Furthermore, the 90-day mortality was significantly decreased by EVT (RR 0.76, 95 % CI 0.65–0.89; heterogeneity I2 = 0 %). There was a higher risk of sICH with EVT (RR 7.77, 95 % CI 2.36–25.59; heterogeneity I2 = 0 %) [47].

### 5.3 Neurosurgery

PCCI, especially cerebellar infarcts, have an elevated risk of swelling and herniation in the posterior fossa. Although medical management (osmotic therapy, hyperventilation, sedation, and optimizing cerebral perfusion pressure) is usually the first line in managing less severe edema, surgery might be the only effective option in patients with significant mass effect. Patients with cerebellar infarction can also develop obstructive hydrocephalus needing ventriculostomy. The risk of upward herniation with ventriculostomy alone can be minimized with conservative drainage of cerebrospinal fluid or subsequent decompression. Decompressive suboccipital craniectomy is recommended if patients deteriorate neurologically or develop signs of brainstem compression despite maximal medical management [48], [49], [50].

## 6 Outcomes

Patients with PCCI arriving at the hospital after 4.5 h or with unknown onset of symptoms may have worse outcomes when compared to ACS patients who present in the extended time window. In an Austrian stroke unit registry, 9208 (4604 patients with ACS and 4604 patients with PCCI) were matched. 477 patients in each group were treated with intravenous thrombolysis. A better 3-month functional outcome was noted in ACS patients when compared to PCCI. Functional outcome was worse in PCCI patients with onset-to-door time >270 min. No significant difference was noted in functional outcomes in patients that were treated with intravenous thrombolysis [51].

In the New England Medical Center Posterior Circulation Registry, basilar artery occlusion, decreased level of consciousness, embolic etiology (instead of atherosclerotic), tetraparesis, and pupil abnormalities were noted to be significant predictors of poor outcomes [52]. Using the BASICS international registry, Greving et al. found that the main predictors of poor outcome in BAO were higher NIHSS, older age, longer time to treatment, absence of hyperlipidemia, and presence of prodromal minor stroke [53]. In the BEYOND-SWIFT registry, Compared to anterior LVO, BAO was associated with increased rates of futile recanalization or a poor outcome despite good reperfusion after EVT (aOR 2.146, 95 %-CI 1.267–3.633). Older age, greater number of passes, intracranial stenting, and greater stroke severity were found to be predictors of futile recanalization in BAO [39]. In a meta-analysis evaluating prognostic factors in PCCI patients after EVT, hypertension, diabetes mellitus, and prior stroke correlated with worse prognosis (interestingly, hyperlipidemia correlated with better prognosis). No outcome differences were noted in patients with atrial fibrillation, smoking, or coronary artery disease [54].

## 7 Conclusion

Given the variable and sometimes vague presentation of PCCI, a thorough history, neurologic exam, and relevant imaging studies are needed to accurately diagnose this condition. The morbidity and mortality of PCCI, especially BAO, remains high. However, recent BAO trials support the efficacy of EVT for appropriately selected patients and should inform appropriate aggression in treatment selection for thrombectomy for BAO patients. Further studies are warranted to study the effectiveness and safety of endovascular therapy for BAO in diverse populations and those presenting with milder deficits (NIHSS <10).

## Ethics approval

Not applicable.

## Funding

Dr. Adam A Dmytriw is supported by the Society of NeuroInterventional Surgery. Dr. Joshua A Hirsch is supported by the Neiman Health Policy Institute. Dr. Robert W Regenhardt is supported by the Society of Vascular and Interventional Neurology, the Heitman Foundation, and the 10.13039/100000002National Institutes of Health.

## CRediT authorship contribution statement

RAA, AAD, RWR, TMLM and JAH determined the content. RAA prepared the first draft. All authors read, provided meaningful revisions, and approved the final manuscript.

## Declaration of Competing Interest

The authors declare the following financial interests/personal relationships which may be considered as potential competing interests: Competing Interests: Dr. Robert W Regenhardt serves on a DSMB for Rapid Medical and is the site PI for trials sponsored by Penumbra and Microvention. Dr. Joshua A Hirsch is a consultant for Medtronic.
